# Association of glucose metabolism and retinopathy signs in non-diabetic individuals in midlife—The Northern Finland Birth Cohort 1966 study

**DOI:** 10.1371/journal.pone.0240983

**Published:** 2020-10-22

**Authors:** Anni Saunajoki, Juha Auvinen, Ville Saarela, Janne-Joonas Uusitalo, Ilmari Leiviskä, Sirkka Keinänen-Kiukaanniemi, M. Johanna Liinamaa, Markku Timonen

**Affiliations:** 1 Center for Life Course Health Research, University of Oulu, Oulu, Finland; 2 Medical Research Center Oulu, Oulu University Hospital and University of Oulu, Oulu, Finland; 3 Healthcare and Social Services of Oulunkaari, Ii, Finland; 4 PEDEGO Research Unit, University of Oulu, Oulu, Finland; 5 Healthcare and Social Services of Selänne, Pyhäjärvi, Finland; 6 Healthcare and Social Services of City of Oulu, Oulu, Finland; Weill Cornell Medical College in Qatar, QATAR

## Abstract

Diabetic retinopathy is a microvascular complication of hyperglycaemia. Little is known about the association of glucose metabolism and retinopathy signs in the non-diabetic middle-aged population. We studied prevalence of retinopathy in a subsample of Northern Finland Birth Cohort study (NFBC1966) of 1809 subjects, at 47 years of age, without previously diagnosed type 2 diabetes and/or blood pressure-lowering medication. All participants underwent clinical evaluations including an oral glucose tolerance test (glucose and insulin values measured at 0, 30, 60 and 120 min) and HbA_1c_. The retinopathy signs were diagnosed by fundus photographs and classified according to the Eurodiab classification scheme. The overall prevalence of newly diagnosed retinopathy was 1.4%. The retinopathy signs were significantly associated with increased 30 min, 1-h and 2-h glucose levels and 2-h insulin level in an OGTT. After adjustment with systolic blood pressure, only 30 min glucose, 1-h glucose and 2-h insulin levels were associated with retinopathy signs. Our findings show the potential role of 30 min and 1-h post-load glucose and 2-h insulin levels as risk factors for retinopathy lesions among the participants without previously diagnosed diabetes or hypertensive medication.

## Introduction

Diabetic retinopathy (DR) is the most common microvascular complication of hyperglycemia and a leading cause of vision impairment globally [1, 2]. Previous studies have identified a number of risk factors for the development of diabetic retinopathy including longer diabetes duration time and higher blood pressure [2, 3]. However, the role of other cardiovascular risk factors such as dyslipidemia [2, 3] and smoking [4, 5] have remained controversial as an etiology for retinopathy. The current diagnostic glucose cut-off points for type 2 diabetes are based on the inflection point of retinopathy prevalence [6], but a linear increase in fasting and post-load glucose levels has been observed years before the onset of type 2 diabetes [7].

It is well known that the retinopathy signs are also seen among the non-diabetic population and uncontrolled blood pressure is the most reported potential risk factor [3, 8–11]. In the non-diabetic population, the prevalence of retinopathy varies from <1% to 17.2% depending on population, age and ophthalmologic methods [3, 8–10, 12, 13]. Although the non-diabetic isolated retinopathy signs may be transient [14], the population-based studies have shown that individuals with signs of retinopathy are at an increased risk of, for example, future type 2 diabetes, hypertension, nephropathy and cardiovascular morbidity [11, 15–18].

Despite this interest, to the best of our knowledge, this is the first study to evaluate the association of glucose metabolism, measured by OGTT (including glucose and insulin levels determined at 0, 30, 60 and 120 min after glucose intake) and HbA_1c_, and retinopathy signs based on ophthalmic examination in the population without previously diagnosed diabetes or hypertensive medication.

## Materials and methods

### Study population

The Northern Finland Birth Cohort 1966 (NFBC1966) is a prospective population-based birth cohort study consisting of individuals whose expected year of birth was 1966. Written informed consent was obtained from all participants. The Ethics Committee of the Northern Ostrobothnia Hospital District in Oulu, Finland approved the study protocol following the principles of the Declaration of Helsinki.

**Fig 1:**
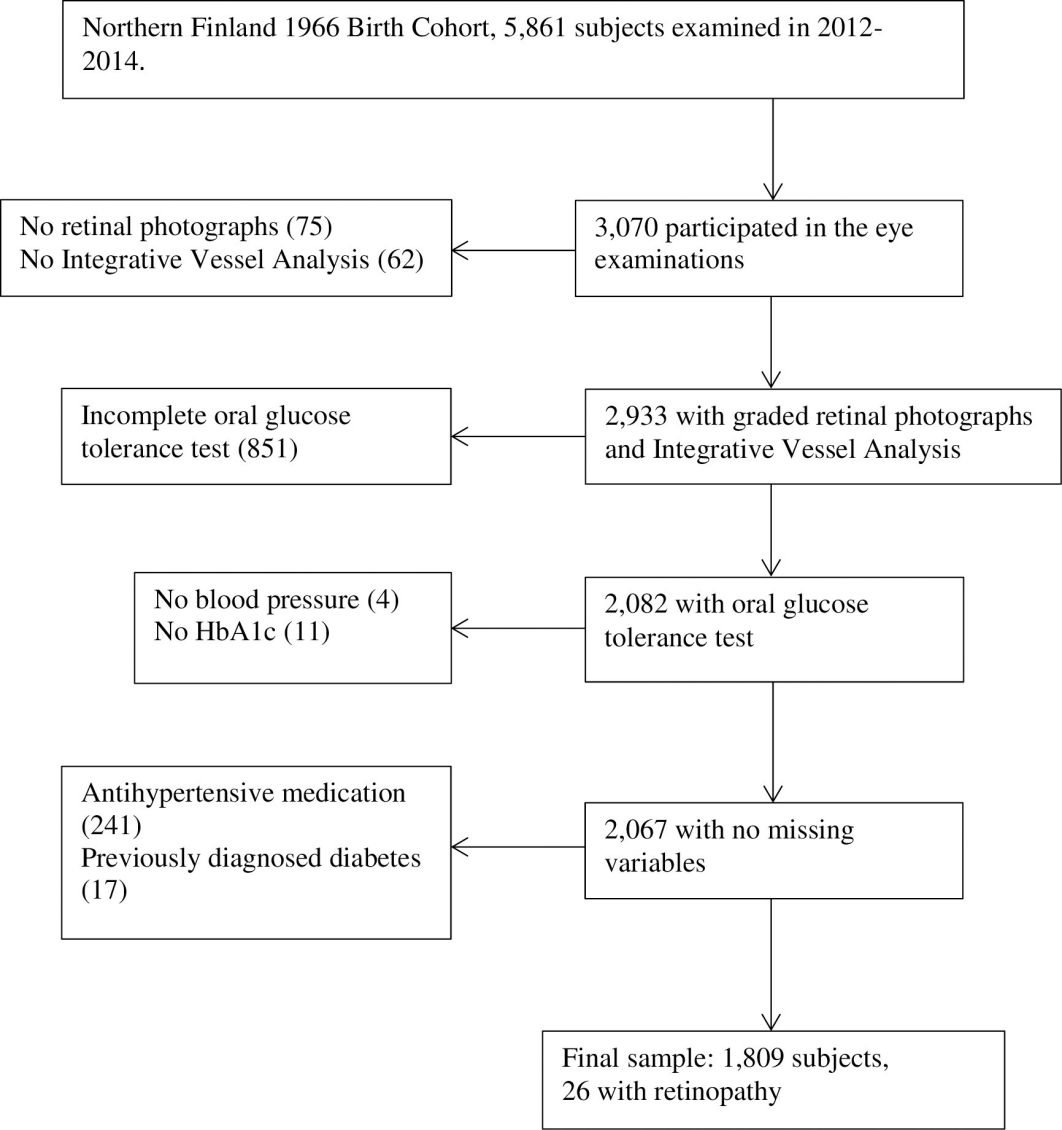
Flowchart of the Northern Finland Birth Cohort 1966 study.

The sample size calculation was based on an estimated retinopathy prevalence in non-diabetic population, and 153 participants were needed to provide a power of 80% at an alpha level of 5%. The original study population consisted of 12,231 participants, 5,861 of whom participated in the 46-year follow-up study carried out in 2012–2014. Participants underwent an oral glucose tolerance test (OGTT) and meticulous ophthalmologic examinations. One half of the 10,321 individuals, who were alive and had known addresses, were randomized to ophthalmologic examinations. Randomisation was based on gender, age, postcode and the month of birth and it was performed using Resampling Stats software (Resampling Stats Inc., Arlington, Virginia, USA) and 3,070 (60%) of the 5,155 randomised subjects attended. Participants with incomplete examinations (more than one missing OGTT time point), previously diagnosed type 2 diabetes and/or who used blood pressure-lowering medication were excluded. A total of 1,809 of 3,070 subject (59%) were included for the final analysis (Fig 1).

### Clinical evaluation

Participants underwent a 75-g OGTT after an overnight fast (12 h). Participants with diagnosed diabetes, medication for diabetes and just before test measured capillary blood glucose level > 8.0 mmol/l were excluded from OGTT. The data on previous diagnosed diabetes and diabetes medications was based on self-reported diagnoses and medications in the questionnaire, hospital registers and medication registers from Social Insurance Institution of Finland. Plasma glucose and serum insulin values were measured at 0, 30, 60 and 120 min after glucose intake. According to WHO criteria, the screen detected diabetes mellitus was diagnosed when fasting plasma glucose was ≥7.0 mmol/l or 2-h plasma glucose was ≥11.1 mmol/l or HbA_1c_ concentration was over 6.5% (48 mmol/mol) [19]. The concentrations of glycated and total hemoglobin were measured using immunochemical assay methods. The HbA_1c_ is reported as percent hemoglobin A1c (NGSP) and mmol/mol. Insulin sensitivity was estimated by HOMA2-IR (the Homeostasis Model Assessment for Insulin Resistance) calculated as (20 × fasting serum insulin) / (fasting plasma glucose– 3.5) × 100, and Matsuda Index (ISI) as 10 000/sqrt (FPG × FSI × (mean OGTT glucose concentration) × (mean OGTT insulin concentration)), where sqrt = square root and FSI = fasting serum insulin [20, 21]. Total cholesterol, high-density lipoprotein and low-density lipoprotein cholesterol, and triglycerides were determined using an enzymatic assay method from fasting blood samples. All the samples were analyzed in testing laboratory (T113), NordLab Oulu, accredited by Finnish Accreditation Service (FINAS) (EN ISO 15189). Seated systolic (SBP) and diastolic blood pressure (DBP) were measured three times in 1-minute periods after 15 minutes of rest with an automated sphygmomanometer (Omron M10, Omron Healthcare, Kyoto, Japan) and a mean of the two lowest systolic values and their diastolic values were used. The height and weight of the participants were measured with light clothing and body mass index (BMI, kg/m^2^) was calculated as weight (kilograms) divided by height (meters) squared.

### Ophthalmic examination and definition of diabetic retinopathy

All participants underwent 45° fundus photographing with a Canon CF-60DSi fundus camera (Canon Inc., Tokyo, Japan) using Neacapture software (Neagen Oy, Oulu, Finland) after pupil dilation with tropicamide 5 mg/ml eye drops. Two photographs were taken from both eyes of each participant centered on the macula and the optic disc. All images were read from both eyes and there were no images that were missing or unreadable. The retinopathy signs were defined by the worse eye. The severity scale of diabetic retinopathy was classified into four categories according to the Eurodiab classification scheme: no retinopathy, mild non-proliferative DR, moderate non-proliferative DR, severe non-proliferative DR and proliferative DR [22]. Both photographic graders were specialists in ophthalmology and were masked to the patients’ identity and clinical diagnosis. A subset of images (100 photographs, representing 5,5% of the whole dataset) was graded by both ophthalmologists to estimate an inter-individual variation (agreement between the readers was 98%, kappa = 0.66 for individuals and kappa = 0.75 for eyes). In our study the image quality was very good, therefore it is not likely that image quality would count for this difference. If there was disagreement between the two graders, the diagnosis of DR was confirmed by the senior ophthalmologist (J.L.).

Retinopathy was considered present when at least one microaneurysm, haemorrhage, or hard exudate was present or in case neovascularization, fibrous proliferation, or laser coagulation scars were seen in the photographs. Hemorrhages or exudates as symptoms of other pathology, such as retinal venous occlusion, were not considered as (diabetic) retinopathy but, if observed, were analyzed separately. Retinal arteriolar and venular calibers were measured from the fundus images as previously described [23]. The optic disc-centered retinal images of the right eye were analyzed by a single grader using a semiautomatic computer-assisted Integrative Vessel Analysis Software (IVAN, University of Wisconsin, Madison, Wisconsin, USA) according to a standardized protocol described earlier [24]. Based on the revised Knudtson-Parr-Hubbard formula [25], three summary variables were created: central retinal arteriolar equivalent (CRAE), central retinal venular equivalent (CRVE) and arteriovenous ratio (AVR) as the ratio of two variables. There were three graders all masked to the participants’ characteristics in current study. The intragrader variability was 4.5% for CRAE and 3.7% for CRVE and the intergrader variability 3.7% for CRAE and 3.0% for CRVE. Moreover, all participants were analyzed by optical coherence tomography (OCT) imaging using spectral‐domain OCT (Cirrus HD‐OCT 4000, Carl Zeiss Meditec AG).

### Statistical methods

The assumption of normally distributed data among variables was checked by Shapiro-Wilk test. The difference between two population means ‘no signs of retinopathy’ and ‘mild or worse retinopathy’ was calculated by using Welch’s t-test or the Mann-Whitney U-test. Welch’s t-test was used for normally distributed variables with unequal variances between groups. Skewed distributions were tested with the Mann-Whitney U-test, which allows testing of the equality of group means in non-normally distributed data. The equality of means in categorical variables including sex, status of diabetes and status of current smoking were assessed with the chi-square test. Logistic regression models were used to estimate the change in the odds for developing retinopathy per unit change in the independent variable. Alternative logistic regression models included a second independent variable—systolic blood pressure. A p-value ≤0.05 was considered significant. The data were analyzed using SPSS software (IBM SPSS Statistics 25.0., IBM Corp., New York).

## Results

**Fig 2:**
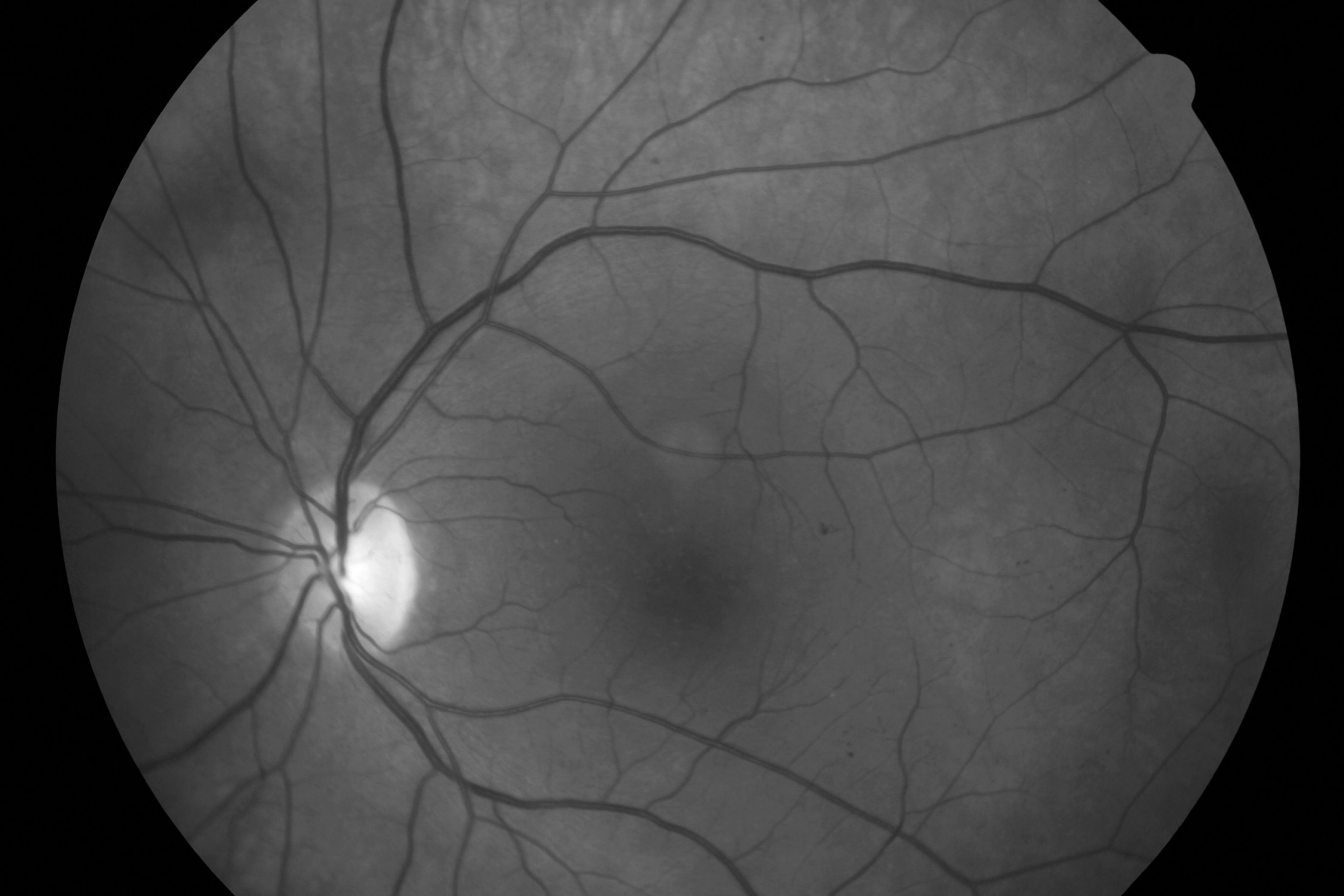
Example of mild non-proliferative retinopathy.

The NFBC1966 included 1,809 participants with ophthalmic and OGTT data available and without previously diagnosed type 2 diabetes or current use of blood pressure medication (Fig 1). The clinical characteristics of the study groups, ‘no retinopathy’ and ‘mild or worse retinopathy’ groups, are shown in Table 1. Mean age did not differ between the groups. Of the 1,809 study participants, 1,783 (98.6%) had no signs of retinopathy and 26 (1.4%) had mild retinopathy. Moderate or severe retinopathy was not observed among any participants. Moreover, among participants with retinopathy signs we did not find any signs of macular telangiestasis type 2 (MACTEL2) in fundus photos, such as retinal graying or dilated venules, nor in OCTs, such as foveal pit temporal enlargement, hyporeflective cavities, pigment plaques or atrophy of neurosensory retina in any of our retinopathy cases. The correlation between the eyes was fair (Kappa 0.283, p = 0.043). Fig 2 shows an example of mild non-proliferative retinopathy.

**Table 1:** Clinical characteristics of participants with and without retinopathy.

	No signs of retinopathy	Mild or worse retinopathy	p value
Study population, n (%)	1783 (98.6)	26 (1.4)	
Age (years)	47.4 (0.9)	47.6 (0.8)	0.168^a^
Sex, n (% male)	718 (40.3)	15 (57.7)	0.072
Screen detected diabetes^b^, n (%)	30 (1.7)	0 (0)	0.505
Current smoker, n (%)	241 (13.9)	4 (16.7)	0.698
BMI (kg/m^2^)	26.3 (4.4)	28.1 (6.8)	0.165^a^
Fasting plasma glucose (mmol/L)	5.4 (0.5)	5.6 (0.4)	0.021^a^
30min post-load glucose (mmol/L)	8.0 (1.6)	8.8 (1.4)	0.006^a^
1h post-load glucose (mmol/L)	7.2 (2.3)	8.6 (2.5)	0.005^a^
2h post-load glucose (mmol/L)	5.8 (1.6)	6.5 (1.4)	0.006^a^
Fasting insulin (μU/ml)	9.4 (8.9)	11.3 (12.4)	0.540^a^
30min serum insulin (μU/ml)	71.2 (48.8)	70.5 (39.3)	0.675^a^
1h serum insulin (μU/ml)	79.1 (59.7)	101.9 (97.2)	0.156^a^
2h serum insulin (μU/ml)	57.0 (51.7)	93.0 (94.9)	0.028^a^
HOMA2 IR	1.2 (0.73)	1.3 (0.8)	0.237^a^
Matsuda index	5.42 (3.08)	3.75 (2.75)	0.185
HbA1c (%)	5.4 (0.35)	5.6 (0.4)	0.112^a^
HbA1c (mmol/mol)	36.0 (4.0)	37.4 (4.3)	0.112^a^
Total cholesterol (mmol/l)	5.31 (0.90)	5.45 (0.94)	0.521
HDL cholesterol (mmol/l)	1.58 (0.38)	1.41 (0.40)	0.017
LDL cholesterol (mmol/l)	3.42 (0.91)	3.57 (0.80)	0.376
Triglyceride (mmol/l)	1.17 (0.77)	1.48 (0.79)	0.006
Systolic blood pressure (mmHg)	121.3 (15.1)	129.2 (20.1)	0.047^a^
Diastolic blood pressure (mmHg)	82.5 (10.2)	87.8 (14.5)	0.101^a^
Retinal arteriolar diameter (μm)	141.5 (14.1)	146.4 (13.4)	0.073
Retinal venular diameter (μm)	218.3 (19.2)	222.8 (20.0)	0.104^a^
Arteriolar-to-venular ratio	0.65 (0.06)	0.66 (0.06)	0.322^a^

Data are mean (SD) or n (%). Continuous variables are compared with Welch’s t-test or the Mann-Whitney U-test (^a^) and categorial variables with the chi-squared test. According to WHO criteria, the screen detected diabetes mellitus (^b^) was diagnosed when fasting plasma glucose was ≥7.0 mmol/l or 2-h plasma glucose was ≥11.1 mmol/l or HbA_1c_ concentration was over 6.5% (48 mmol/mol). Subjects with previously diagnosed type 2 diabetes were excluded.

Subjects with retinopathy signs had significantly higher fasting and post-load glucose values, 2-h serum insulin level in an OGTT, systolic blood pressure, triglycerides and lower HDL cholesterol (p<0.05). In contrast, we did not find a statistically significant association between the presence of retinopathy and diastolic blood pressure, BMI, smoking status, HbA_1c_, fasting insulin, 30 min insulin, 1-h insulin, HOMA2-IR, Matsuda Index, total cholesterol or LDL cholesterol. Furthermore, retinal arteriolar and venular widening or arteriolar-to-venular ratio were not either associated with microaneurysms.

Table 2 presents the association of retinopathy with hyperglycemia and hypertension in persons without previous type 2 diabetes. A higher level of fasting plasma glucose (OR 1.79, 95% Cl 1.00–3.21, p = 0.05), 30 min glucose (OR 1.38, 95% Cl 1.10–1.74, p < 0.01), 1-h glucose (OR 1.24, 95% Cl 1.08–1.43, p < 0.01), and 2-h glucose in an OGTT (OR 1.22, 95% Cl 1.03–1.43, p < 0.05), were significantly associated with the presence of retinopathy signs. Higher systolic blood pressure (OR 1.03, 95% Cl 1.01–1.05, p < 0.01) and OGTT 2-h insulin (OR 1.007, 95% Cl 1.001–1.011, p < 0.01) were also associated with an excess risk of DR. In contrast, such significant associations were not observed between the retinopathy and fasting insulin, 30 min insulin, 1-h insulin, HbA_1c_, HOMA2-IR or Matsuda index. After adjustment with systolic blood pressure, a higher level of 30 min glucose (OR 1.31, 95% CI 1.03–1.66, p < 0.05), 1-h glucose (OR 1.20, 95% CI 1.03–1.39, p < 0.05) and 2-h insulin (OR 1.006, 95% CI 1.001–1.010, p < 0.05) were associated with DR.

**Table 2:** Association of retinopathy with hyperglycaemia and hypertension.

	Unadjusted			Model I		
	OR	95% CI	P	OR	95% CI	P
Fasting plasma glucose	1.79	(1.00 to 3.21)	0.050	1.53	(0.81 to 2.89)	0.189
30 min glucose	1.38	(1.10 to 1.74)	0.006	1.31	(1.03 to 1.66)	0.029
1-h glucose	1.24	(1.08 to 1.43)	0.002	1.20	(1.03 to 1.39)	0.017
2-h glucose	1.22	(1.03 to 1.43)	0.021	1.17	(0.97 to 1.40)	0.098
Fasting insulin	1.01	(0.99 to 1.04)	0.289	1.01	(0.98 to 1.04)	0.515
30 min insulin	1.00	(0.99 to 1.01)	0.944	1.00	(0.99 to 1.01)	0.660
1-h insulin	1.005	(1.00 to 1.009)	0.059	1.003	(0.998 to 1.008)	0.196
2-h insulin	1.007	(1.001 to 1.011)	0.001	1.006	(1.001 to 1.010)	0.014
HbA_1c_	1.09	(1.00 to 1.19)	0.065	1.07	(0.98 to 1.17)	0.143
HOMA2-IR	1.25	(0.79 to 1.98)	0.351	1.09	(0.66 to 1.92)	0.737
Matsuda Index	0.91	(0.78 to 1.06)	0.211	0.95	(0.82 to 1.11)	0.541
Systolic blood pressure	1.03	(1.01 to 1.05)	0.009	-	-	-

Data are odds ratios i.e. increase in odds for developing retinopathy per unit change in the variables and their 95% confidence intervals. Model I is adjusted for systolic blood pressure.

**Fig 3:**
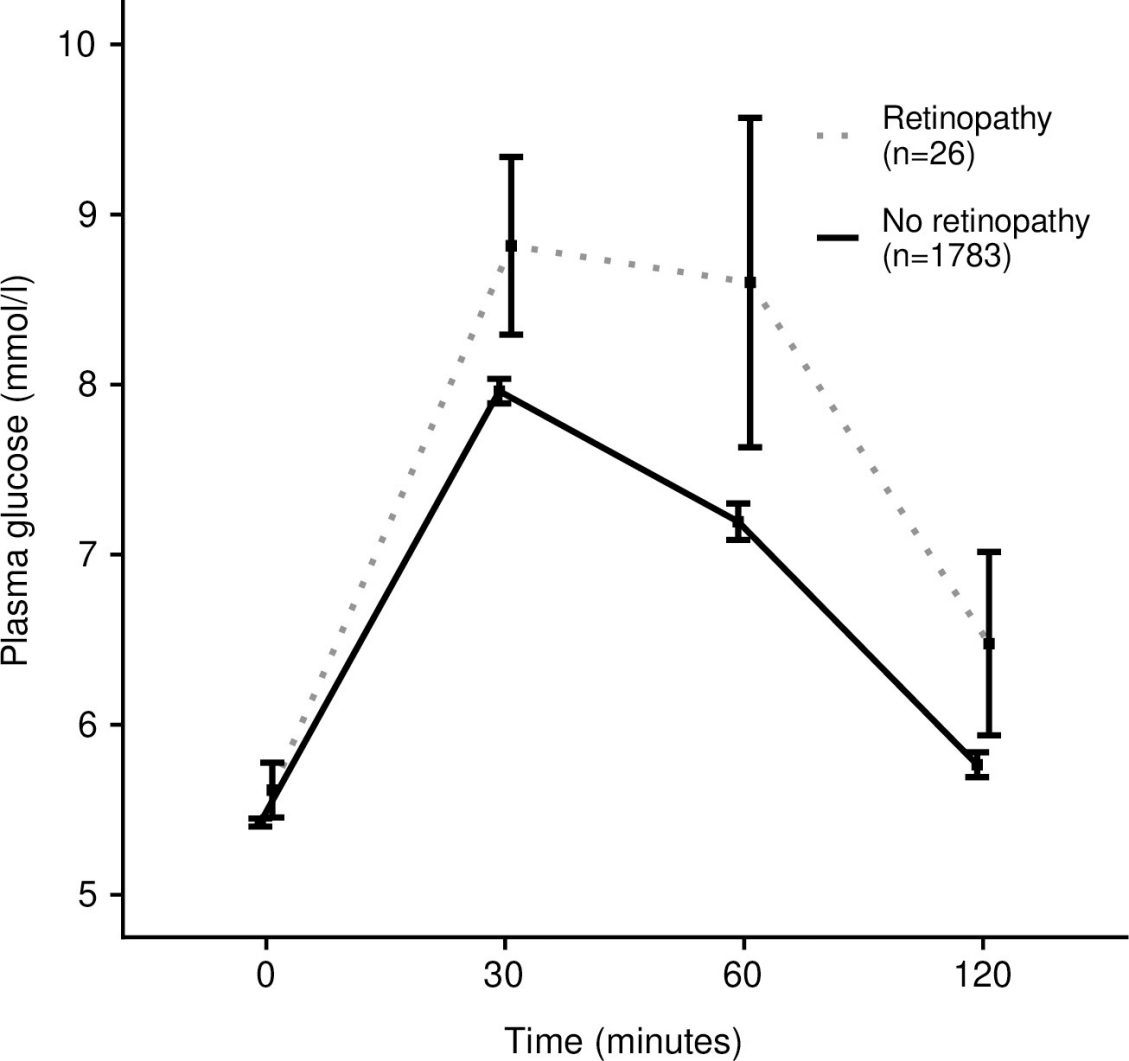
Change in plasma glucose level (mmol/l) during the oral glucose tolerance test (OGTT) of two study groups; no retinopathy and retinopathy.

**Fig 4:**
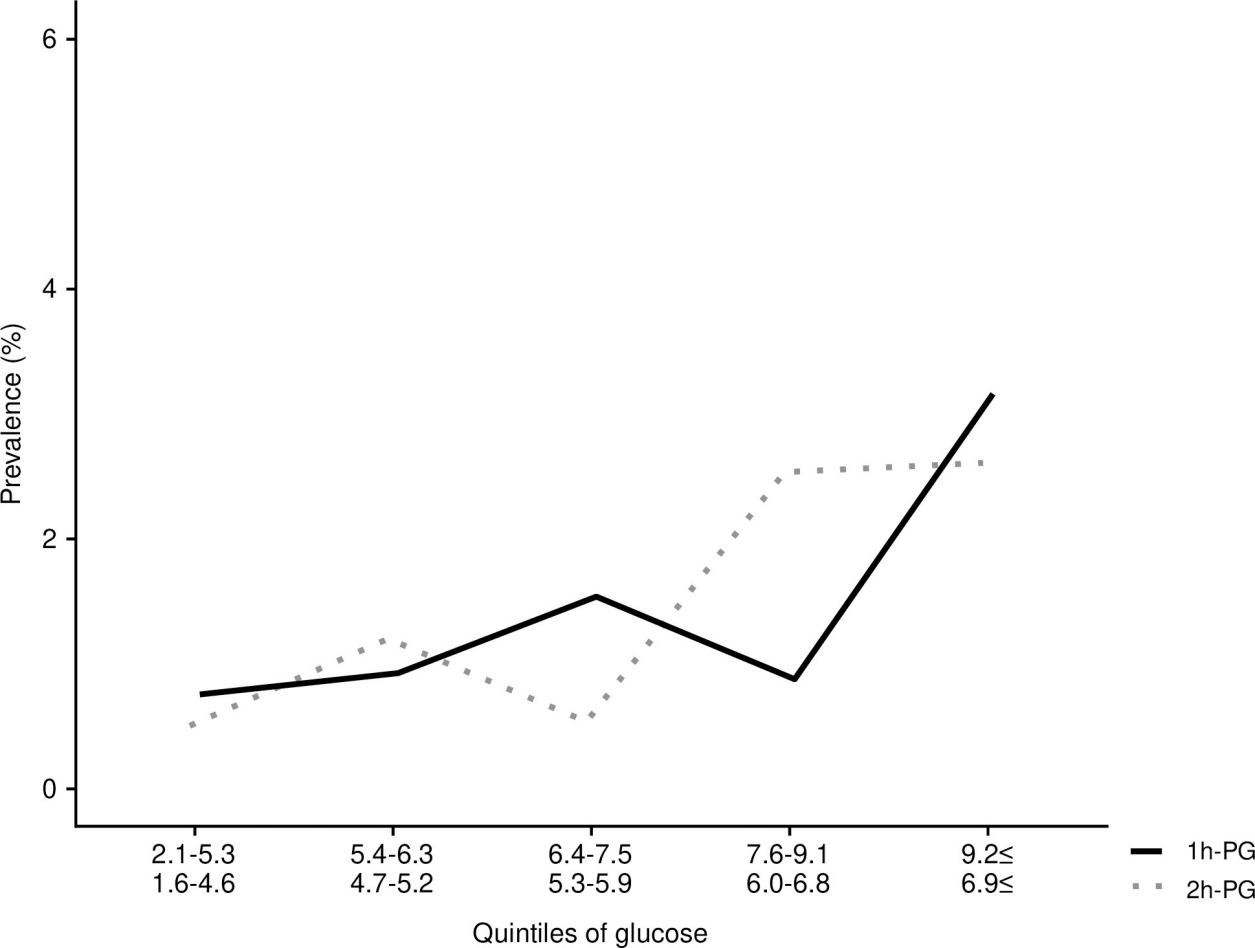
Retinopathy prevalence (%) of 1-h glucose and 2-h glucose concentrations by glucose quantiles. Retinopathy prevalence is based on fundus photographs. The lowest glucose values of each quintiles are presented in the figure.

Fig 3 presents changes in plasma glucose levels at four different time points during the OGTT. Participants with higher plasma glucose concentrations were significantly more likely to have microaneurysms (Fig 3). To further illustrate this result, Fig 4 shows that the prevalence of retinopathy increases across quantiles of each glycemic measure. The prevalence was gradually increased when the OGTT 1-h glucose was within the range 7.6–9.1 mmol/l and 2-h glucose 5.3–6.0 mmol/l (Fig 4).

## Discussion

In this middle-aged birth cohort population, without previously diagnosed diabetes or hypertensive medication, the overall prevalence of newly diagnosed retinopathy was 1.4%. The retinopathy signs were associated with increased glucose and 2-h insulin levels in OGTT as well as systolic blood pressure, triglycerides and lower HDL cholesterol. After adjustment with systolic blood pressure, 30 min glucose, 1-h glucose and 2-h insulin were independently associated with retinopathy. The number of subjects with retinopathy was too low to analyze the diagnostic glucose threshold value for retinopathy signs, although the prevalence of retinopathy was gradually increased in the highest quintiles of 1-h and 2-h glucose.

In our study, the prevalence of non-diabetic retinopathy signs was relatively low 1.4%. According to the previous studies, the prevalence has been varying between <1% and 17.2% [3, 8–10, 12, 13]. In the Multi-Ethnic Study of Atherosclerosis, the ethnicity influenced in the prevalence of retinopathy being highest in Chinese (17.2%) and the lowest in white population (11.9%) [8]. In contrast, in the Framingham study the prevalence of the retinopathy was 0.8%. Compared to the other studies, the retinopathy signs were evaluated by direct ophthalmoscopy which probably underestimates the actual prevalence [13]. Moreover, non-diabetic retinopathy has been more prevalent among participants with older age [9], higher glycemic status [3, 9, 10, 12] or long-term uncontrolled blood pressure [3, 11]. So, the disparity of the non-diabetic retinopathy prevalence can be explained by a combination of these characteristic and methodological differences between the studies.

Important finding was that 30 min and 1-h post-load glucose and 2-h insulin levels were independently associated with retinopathy. The usefulness of elevated 1-h glucose to predict retinopathy was previously seen in a population-based study, but the retinopathy was determined using direct ophthalmoscopy [26]. Several studies have estimated the association of hyperglycaemia and development of non-diabetic retinopathy signs determining the retinopathy also by fundus photography [3, 12, 27–29]. In contrast to ours, they evaluated the association of retinopathy signs only to fasting, 2-h post-load glucose levels or HbA1c. Thus, to the best of our knowledge, there is no comparable studies available.

The results of this study found that subjects with retinopathy signs did not have statistically wider retinal arteriole and venular calibers than in the non-retinopathy group. A number of population-based studies have previously shown the association with retinal vascular changes and increased risk of developing type 2 diabetes. The retinal arteriolar narrowing has been suggested to predict the impaired fasting glucose and incident diabetes in the Rotterdam study from Netherlands (n = 2,309, 55 years or older) [30]. In contrast, the Blue Mountain Eye Study from Australia (n = 3,654, 49 years or older) and the Multi-Ethnic Study of Atherosclerosis including whites, blacks, Hispanics, and Chinese (n = 4,585, 45 years or older) demonstrated the wider venular calibers to predict the risk [31, 32]. Ethnicity, hypertension and age may explain the variation of these results.

The pathophysiological mechanism and clinical significance of non-diabetic retinopathy signs are not fully understood. The combination of factors, for example hyperglycemia [10, 12], insulin resistance [33], hypertension [3] and systematic inflammation [34, 35], has assumed to cause the early retinal microvascular damage. In our study, 30 min and 1-h post-load glucose and 2-h insulin were independently associated with retinopathy, however, we did not confirm such association with insulin resistance indicators. One explanation for this discrepancy is that the retinopathy prevalence was too low (26 patients) making the further analyses underpowered. Furthermore, non-diabetic retinopathy signs have been related to increased incidence of complications, such as stroke [18], congestive heart failure [17] and renal dysfunction [15]. Due to the risk profile, participants with retinopathy signs should be more closely monitored.

A few limitations of this study could be noted. The cross-sectional study design does not give any information on the causality. Also, the sample size of subjects with retinopathy was relatively small and that may have limited our capacity for the further analyses of, for example, the diagnostic glucose threshold value for retinopathy signs. It is also noteworthy that our analyses included some participants with untreated hypertension. To diminish the potential impact of blood pressure, we adjusted the results with systolic blood pressure. The strength of the present study is the use of standardized retinopathy grading with golden standard protocols and macular OCTs supporting the validity of our findings. Moreover, intensive OGTT had four time points and both glucose and insulin measurements as well as detailed information about the general health of participants.

In conclusion, the data from this study show a 1.4% prevalence of newly diagnosed retinopathy in the middle-aged population without previously diagnosed diabetes or hypertensive medications. Only 30 min glucose, 1-h glucose and 2-h insulin were associated with retinopathy signs, after controlling with systolic blood pressure. Our findings show the potential role of these increased post-load glucose and insulin levels as risk factors for retinopathy development. However, prospective studies are required to evaluate the progression of these early diabetic changes and glycemic status.

## References

[1] Klein BE. Overview of epidemiologic studies of diabetic retinopathy. Ophthalmic Epidemiol. 2007;14: 179–183. 10.1080/09286580701396720 17896294

[2] Yau JW, Rogers SL, Kawasaki R, Lamoureux EL, Kowalski JW, Bek T, et al Global prevalence and major risk factors of diabetic retinopathy. Diabetes Care. 2012;35: 556–564. 10.2337/dc11-1909 22301125 PMC3322721

[3] van Leiden HA, Dekker JM, Moll AC, Nijpels G, Heine RJ, Bouter LM, et al Blood pressure, lipids, and obesity are associated with retinopathy: the hoorn study. Diabetes Care. 2002;25: 1320–1325. 10.2337/diacare.25.8.1320 12145228

[4] Moss SE, Klein R, Klein BE. Association of cigarette smoking with diabetic retinopathy. Diabetes Care. 1991;14: 119–126. 10.2337/diacare.14.2.119 2060413

[5] Cunningham ET, Adamis AP, Altaweel M, Aiello LP, Bressler NM, D'Amico DJ, et al A phase II randomized double-masked trial of pegaptanib, an anti-vascular endothelial growth factor aptamer, for diabetic macular edema. Ophthalmology. 2005;112: 1747–1757. 10.1016/j.ophtha.2005.06.007 16154196

[6] [Anonymous]. Report of the Expert Committee on the Diagnosis and Classification of Diabetes Mellitus. Diabetes Care. 1997;20: 1183–1197. 10.2337/diacare.20.7.1183 9203460

[7] Tabak AG, Jokela M, Akbaraly TN, Brunner EJ, Kivimaki M, Witte DR. Trajectories of glycaemia, insulin sensitivity, and insulin secretion before diagnosis of type 2 diabetes: an analysis from the Whitehall II study. Lancet. 2009;373: 2215–2221. 10.1016/S0140-6736(09)60619-X 19515410 PMC2726723

[8] Ojaimi E, Nguyen TT, Klein R, Islam FM, Cotch MF, Klein BE, et al Retinopathy signs in people without diabetes: the multi-ethnic study of atherosclerosis. Ophthalmology. 2011;118: 656–662. 10.1016/j.ophtha.2010.08.007 21055817 PMC3045651

[9] Kawasaki R, Wang JJ, Rochtchina E, Taylor B, Wong TY, Tominaga M, et al Cardiovascular risk factors and retinal microvascular signs in an adult Japanese population: the Funagata Study. Ophthalmology. 2006;113: 1378–1384. 10.1016/j.ophtha.2006.02.052 16877076

[10] Bhargava M, Cheung CY, Sabanayagam C, Huang L, Lamoureux EL, Wang JJ, et al Prevalence and risk factors for retinopathy in persons without diabetes: the Singapore Indian Eye Study. Acta Ophthalmol. 2014;92: e602–9. 10.1111/aos.12446 24894034

[11] Klein R, Myers CE, Lee KE, Klein BE. 15-Year Cumulative Incidence and Associated Risk Factors for Retinopathy in Nondiabetic Persons. Arch Ophthalmol. 2010;128: 1568–1575. 10.1001/archophthalmol.2010.298 21149781 PMC3058844

[12] Rajala U, Laakso M, Qiao Q, Keinanen-Kiukaanniemi S. Prevalence of retinopathy in people with diabetes, impaired glucose tolerance, and normal glucose tolerance. Diabetes Care. 1998;21: 1664–1669. 10.2337/diacare.21.10.1664 9773727

[13] Leibowitz HM, Krueger DE, Maunder LR, Milton RC, Kini MM, Kahn HA, et al The Framingham Eye Study monograph: An ophthalmological and epidemiological study of cataract, glaucoma, diabetic retinopathy, macular degeneration, and visual acuity in a general population of 2631 adults, 1973–1975. Surv Ophthalmol. 1980;24: 335–610. 7444756

[14] Cugati S, Cikamatana L, Wang JJ, Kifley A, Liew G, Mitchell P. Five-year incidence and progression of vascular retinopathy in persons without diabetes: the Blue Mountains Eye Study. Eye (Lond). 2006;20: 1239–1245. 16167076 10.1038/sj.eye.6702085

[15] Edwards MS, Wilson DB, Craven TE, Stafford J, Fried LF, Wong TY, et al Associations between retinal microvascular abnormalities and declining renal function in the elderly population: the Cardiovascular Health Study. Am J Kidney Dis. 2005;46: 214–224. 10.1053/j.ajkd.2005.05.005 16112039

[16] Wong TY, Klein R, Sharrett AR, Nieto FJ, Boland LL, Couper DJ, et al Retinal microvascular abnormalities and cognitive impairment in middle-aged persons: the Atherosclerosis Risk in Communities Study. Stroke. 2002;33: 1487–1492. 10.1161/01.str.0000016789.56668.43 12052979

[17] Wong TY, Rosamond W, Chang PP, Couper DJ, Sharrett AR, Hubbard LD, et al Retinopathy and risk of congestive heart failure. JAMA. 2005;293: 63–69. 10.1001/jama.293.1.63 15632337

[18] Wong TY, Klein R, Couper DJ, Cooper LS, Shahar E, Hubbard LD, et al Retinal microvascular abnormalities and incident stroke: the Atherosclerosis Risk in Communities Study. Lancet. 2001;358: 1134–1140. 10.1016/S0140-6736(01)06253-5 11597667

[19] Inzucchi SE. Clinical practice. Diagnosis of diabetes. N Engl J Med. 2012;367: 542–550. 10.1056/NEJMcp1103643 22873534

[20] Wallace TM, Levy JC, Matthews DR. Use and abuse of HOMA modeling. Diabetes Care. 2004;27: 1487–1495. 10.2337/diacare.27.6.1487 15161807

[21] Matsuda M, DeFronzo RA. Insulin sensitivity indices obtained from oral glucose tolerance testing: comparison with the euglycemic insulin clamp. Diabetes Care. 1999;22: 1462–1470. 10.2337/diacare.22.9.1462 10480510

[22] Aldington SJ, Kohner EM, Meuer S, Klein R, Sjolie AK. Methodology for retinal photography and assessment of diabetic retinopathy: the EURODIAB IDDM complications study. Diabetologia. 1995;38: 437–444. 10.1007/BF00410281 7796984

[23] Geneid M, Kettunen J, Nuuttila I, Lintonen T, Uusitalo JJ, Saarela V, et al Relationship between retinal vessel diameter with both retinal nerve fibre layer thickness and optic nerve head parameters in middle-aged Caucasians: the Northern Finland Birth Cohort Eye study. Acta Ophthalmol. 2019;97: 532–538. 10.1111/aos.13992 30537339 PMC6767424

[24] Wong TY, Knudtson MD, Klein R, Klein BE, Meuer SM, Hubbard LD. Computer-assisted measurement of retinal vessel diameters in the Beaver Dam Eye Study: methodology, correlation between eyes, and effect of refractive errors. Ophthalmology. 2004;111: 1183–1190. 10.1016/j.ophtha.2003.09.039 15177969

[25] Knudtson MD, Lee KE, Hubbard LD, Wong TY, Klein R, Klein BE. Revised formulas for summarizing retinal vessel diameters. Curr Eye Res. 2003;27: 143–149. 10.1076/ceyr.27.3.143.16049 14562179

[26] Paddock E, Looker HC, Piaggi P, Knowler WC, Krakoff J, Chang DC. One-Hour Plasma Glucose Compared With 2-Hour Plasma Glucose in Relation to Diabetic Retinopathy in American Indians. Diabetes Care. 2018. 10.2337/dc17-1900 PMC5961391 29622542

[27] Akhter A, Fatema K, Ahmed SF, Afroz A, Ali L, Hussain A. Prevalence and associated risk indicators of retinopathy in a rural Bangladeshi population with and without diabetes. Ophthalmic Epidemiol. 2013;20: 220–227. 10.3109/09286586.2013.809770 23865602

[28] Tapp RJ, Shaw JE, Harper CA, de Courten MP, Balkau B, McCarty DJ, et al The prevalence of and factors associated with diabetic retinopathy in the Australian population. Diabetes Care. 2003;26: 1731–1737. 10.2337/diacare.26.6.1731 12766102

[29] Cheng YJ, Gregg EW, Geiss LS, Imperatore G, Williams DE, Zhang X, et al Association of A1C and fasting plasma glucose levels with diabetic retinopathy prevalence in the U.S. population: Implications for diabetes diagnostic thresholds. Diabetes Care. 2009;32: 2027–2032. 10.2337/dc09-0440 19875604 PMC2768189

[30] Ikram MK, Janssen JA, Roos AM, Rietveld I, Witteman JC, Breteler MM, et al Retinal vessel diameters and risk of impaired fasting glucose or diabetes: the Rotterdam study. Diabetes. 2006;55: 506–510. 10.2337/diabetes.55.02.06.db05-0546 16443787

[31] Kifley A, Wang JJ, Cugati S, Wong TY, Mitchell P. Retinal vascular caliber, diabetes, and retinopathy. Am J Ophthalmol. 2007;143: 1024–1026. 10.1016/j.ajo.2007.01.034 17524767

[32] Nguyen TT, Wang JJ, Sharrett AR, Islam FM, Klein R, Klein BE, et al Relationship of retinal vascular caliber with diabetes and retinopathy: the Multi-Ethnic Study of Atherosclerosis (MESA). Diabetes Care. 2008;31: 544–549. 10.2337/dc07-1528 18070990

[33] Bao YK, Yan Y, Wilson B, Gordon MO, Semenkovich CF, Rajagopal R. Association of Retinopathy and Insulin Resistance: NHANES 2005–2008. Curr Eye Res. 2019: 1–4. 10.1080/02713683.2019.1659977 PMC6980426 31460803

[34] Rubsam A, Parikh S, Fort PE. Role of Inflammation in Diabetic Retinopathy. Int J Mol Sci. 2018;19: 10.3390/ijms19040942 29565290 PMC5979417

[35] Tang J, Kern TS. Inflammation in diabetic retinopathy. Prog Retin Eye Res. 2011;30: 343–358. 10.1016/j.preteyeres.2011.05.002 21635964 PMC3433044

